# Dual Roles of IL-27 in Cancer Biology and Immunotherapy

**DOI:** 10.1155/2017/3958069

**Published:** 2017-02-01

**Authors:** Marina Fabbi, Grazia Carbotti, Silvano Ferrini

**Affiliations:** Laboratory of Biotherapy, IRCCS AOU San Martino-IST, Istituto Nazionale per la Ricerca sul Cancro, 16132 Genoa, Italy

## Abstract

IL-27 is a pleiotropic two-chain cytokine, composed of EBI3 and IL-27p28 subunits, which is structurally related to both IL-12 and IL-6 cytokine families. IL-27 acts through a heterodimer receptor consisting of IL-27R*α* (WSX1) and gp130 chains, which mediate signaling predominantly through STAT1 and STAT3. IL-27 was initially reported as an immune-enhancing cytokine that supports CD4^+^ T cell proliferation, T helper (Th)1 cell differentiation, and IFN-*γ* production, acting in concert with IL-12. However, subsequent studies demonstrated that IL-27 displays complex immune-regulatory functions, which may result in either proinflammatory or anti-inflammatory effects in relationship to the biological context and experimental models considered. Several pieces of evidence, obtained in preclinical tumor models, indicated that IL-27 has a potent antitumor activity, related not only to the induction of tumor-specific Th1 and cytotoxic T lymphocyte (CTL) responses but also to direct inhibitory effects on tumor cell proliferation, survival, invasiveness, and angiogenic potential. Nonetheless, given its immune-regulatory functions, the effects of IL-27 on cancer may be dual and protumor effects may also occur. Here, we will summarize IL-27 biological activities and its functional overlaps with the IFNs and discuss its dual role in tumors in the light of potential applications to cancer immunotherapy.

## 1. Introduction

Cytokines such as IFN-*α* and IL-2 represent milestones in the history of cancer immunotherapy [1] and are still used as anticancer agents in different tumors. Importantly, immunotherapy with IL-2 has provided the proof of principle that harnessing the immune response may result in the regression of bulky metastatic lesions and may lead to long-term remissions [2]. Nonetheless, the response rates to IL-2 in metastatic renal cancer and melanoma patients are small, although long-lasting complete remissions have been observed. In more recent years, clinical studies of immune checkpoint blockers, such as the anti-CTLA-4 mAb ipilimumab [3], and the anti-PD-1 mAbs nivolumab [4] and pembrolizumab [5] have shown unprecedented results in metastatic cancer patients. Indeed, these agents can awaken a silenced antitumor T cell response, leading to regression of metastatic cancers and long-lasting remissions in a significant fraction of patients [6]. The presence of abundant tumor-infiltrating CD8^+^ T cells correlates with the expression of the PD-1 ligand PD-L1 in the same tumor areas and with increased clinical response rates to immune checkpoint blockers [7].

These favorable tumor characteristics indicate the preexistence of an antitumor CTL response. However, this response results in a local production of IFN-*γ*, which upregulates PD-L1 expression in the surrounding tumor area, thus resulting in an “adaptive immune resistance” [8]. PD-L1 expressed on tumor cells upon engagement with PD-1 at the cell surface of previously activated CTLs provides an inhibitory signal, which suppresses their functions and may induce apoptosis [9].

Regarding the development of additional agents for cancer immunotherapy, several other molecules have shown promising results in preclinical studies. Among them, several cytokines (e.g., IL-15, IL-21, and IL-18) have shown impressive results in mouse models and have also been tested in clinical trials [reviewed in [10–15]]. However, response rates in clinical studies of cytokines, including, for example, IL-18 [16] and IL-21 [17], have been considerably lower than those achieved with immune checkpoint blockers and not superior to those obtained with standard therapies. These disappointing results may be due to several reasons, such as the multiplicity of cellular targets and the pleiotropic effects of these cytokines. Indeed, most, if not all, cytokines have dual effects on the immune system as they exert on one hand immune-stimulating function, and, on the other hand, they trigger immune-regulatory loops. For example, IL-21 mediates CTL and secondary cytokine/chemokine-mediated tumor rejection [18] and may act as a mediator of autoimmunity [19]. However, it also induces IL-10 expression in T and B cells, resulting in immune-regulatory activities [20]. The dual effects of IL-21 [13] and many other cytokines reflect the need of control mechanisms of their activity, which preserve the host from a potentially damaging, excessive immune stimulation.

IL-27 is a cytokine of the IL-12 family [47] and has shown impressive antitumor effects in different cancer models. Therefore, IL-27 has been proposed as a potential new agent to be explored in cancer immunotherapy studies [reviewed in [48]]. However, it displays pleiotropic functions, which comprise both immune-enhancing and immune-regulatory effects [49]. Here we will address the dual role of IL-27 in immune regulation and cancer and its functional similarities with IFN-*γ*. Indeed, recent data suggest a broad functional overlap between IL-27 and IFN-*γ*, in relationship to the common usage of the STAT1 signaling pathway [25].

## 2. IL-27 Is a Member of the IL-12 and IL-6 Cytokine Families

**Figure 1:**
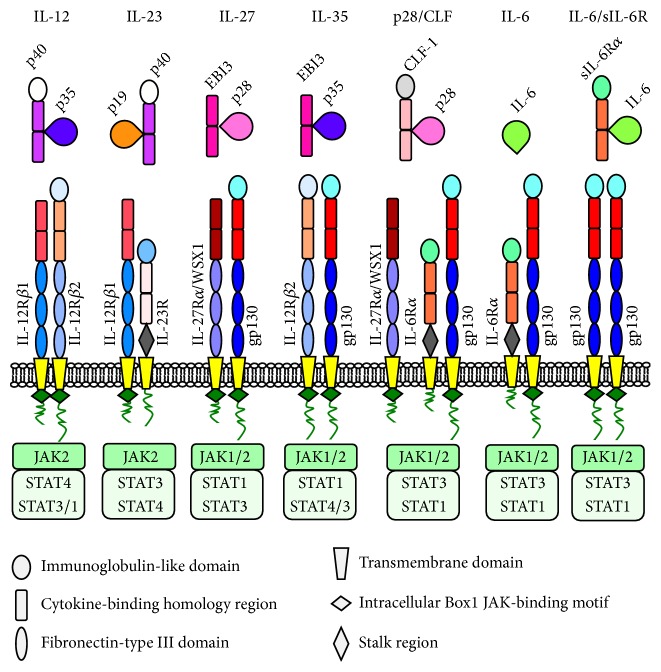
Examples of cytokine and cytokine receptor chain sharing among members of the IL-12 and IL-6 cytokine families. The main signaling pathways are also indicated.

IL-27 is a member of a family of dimer cytokines, which also includes IL-12, IL-23, and IL-35 [50, 51]. The members of this family bind to heterodimeric receptors and may share chain components in both cytokines and receptor chains (Figure 1). In addition, IL-27 shares structural similarities with the IL-6 cytokine family. In fact, it is composed of the IL-27p28 chain and the Epstein-Barr Virus-induced gene 3 (EBI3) [52], which is structurally related to the IL-6R and the IL-12p40 chain [53]. IL-27p28 is a four-*α* helix bundle protein, a structure common to many cytokines, including IL-6, IL-12p35, and IL-23p19. IL-27p28 was discovered through the computer search for proteins with an IL-6-like structure. Its association with EBI3 was evidenced through the search for a partner protein among various nonsignaling receptors of the IL-6 family that would allow efficient secretion and biological activity [52]. EBI3 can also pair with IL-12p35 to generate IL-35, which is involved in Treg-mediated immune-regulatory functions [54]. IL-35 also induces Breg cells, which produce IL-35 and IL-10 and confer protection from experimental autoimmune uveitis (EAU), inhibiting pathogenic Th17 cells [55]. In addition, mice with B cell selective deficiency of p35 or EBI3 developed exacerbated forms of EAE, while these mice were more resistant to infection with a pathogenic* Salmonella* strain [56]. Recent findings indicate that EBI3 makes a new IL-12 family member by pairing with IL-23p19 to form IL-39, which is produced by LPS-stimulated B cells and B cells of lupus-like mice [57]. In addition, IL-27p28 can partner with the Cytokine-Like Factor-1 (CLF-1) to form a functional heterodimer, which regulates T and NK cell activity through IL-6R components [58].

IL-27 binds to a heterodimeric cell surface receptor, which consists of the gp130 and the IL-27R*α*, also named WSX1/TCCR [52, 59]. Gp130 is a common receptor subunit in several other cytokine receptors including the receptors for IL-35 [60], IL-6, Oncostatin M (OSM), Leukemia Inhibitory Factor (LIF), Cardiotrophin 1 (CT-1), Ciliary Neurotrophic Factor (CNTF), and IL-11 [61]. However, there are contradicting reports on the IL-35 receptor complex composition, which has been reported as IL-12R*β*2 homodimer, gp130 homodimer, IL-12R*β*2/gp130, and IL-12R*β*2/WSX1 [55, 60]. IL-27R*α* may also form a trimeric complex with gp130 and IL-6R*α* to bind the IL-27p28/CLF-1 heterodimer [58]. However, it should be noted that cells from IL-27R*α*-deficient mice do not respond to IL-27 but can still respond to IL-27p28/CLF-1, indicating that this heterodimer cytokine can also bind to different receptor complexes [62]. This finding suggests some levels of plasticity within the IL-6/IL-12 cytokine and receptor families.

As the gp130 subunit is ubiquitously expressed, the cell-specificity of IL-27 activity is restricted by the coexpression of IL-27R*α*. IL-27R*α* is present on T, B, and NK lymphocytes, neutrophils, monocytes, and mast cells, although it is found at lower levels in macrophages, hepatocytes, keratinocytes, and endothelial cells [63]. The broad activity of IL-27 on different types of human cancer cells implies IL-27R*α* expression in these cells, as was formally demonstrated in different reports [21, 22, 30, 42, 64].

Regarding the cell source of IL-27, this cytokine is mainly produced by cells of myeloid origin such as monocytes, macrophages, dendritic cells, and microglial cells, in response to stimuli acting through Toll-like receptors [65] or TNF-R-family members, for example, CD40L [66]. Also, type-I IFN and IFN-*γ* can promote IL-27 expression, acting in cooperation with TLR signaling [67, 68]. A recent study identified a novel subset of malaria antigen-specific, IL-27-producing regulatory CD4^+^ T cells in mice infected with* Plasmodium berghei* ANKA. These cells expressed a FoxP3^−^CD11a^+^CD49d^+^ phenotype and were distinct from IL-10-producing type-1 regulatory (Tr1) cells [69].

Finally, IL-27p28 may be secreted independently from EBI3 as IL-27p28 monomers or homodimers, named IL-30. It should be noted that IL-27p28 has been shown to be secreted from cells on its own only in mice, so far. It has been reported that IL-30 may act as cytokine receptor antagonist and limit cytokine signaling [70, 71]. However, another report showed that murine recombinant IL-30 expressed in bacteria and properly refolded did not interfere with IL-6- or IL-27-induced signaling and may act as an agonist through the mouse and human IL-6R [72]. It is well known that IL-6 can bind to soluble (s)IL-6R*α* and that this complex can mediate transsignaling in gp130 expressing cells independently from membrane IL-6R*α* [73]. This knowledge led to the development of the artificial designer cytokine hyper-IL-6 whose structure mimics natural IL-6/sIL-6R*α* complexes [74]. Similarly, the IL-27p28 monomer can bind sIL-6R*α* and mediate transsignaling in cells expressing gp130, and its activity is blocked by soluble (s)gp130 [72]. Interestingly, sgp130 cannot block IL-27 signaling [75], but it can block IL-11/sIL-11R signaling [76], suggesting that sgp130 can only block soluble cytokine/cytokine receptor complexes that signal through gp130 homodimers but not heterodimers.

IL-27 binding with the gp130/IL-27R*α* complex activates the JAK/STAT signaling pathway, which mainly involves STAT1 and STAT3 phosphorylation [77]. STAT3 activation by IL-27 induces the expression of SOCS3, which inhibits further IL-27 signaling in a negative feedback loop, through inhibition of JAK activity [78]. Of note, the IL-27R*α* ectodomain can be released from monocyte-derived DC and, upon mitogen stimulation, from T cells through a metalloprotease-dependent mechanism. Soluble IL-27R*α* is found in the serum, can bind IL-27, and act as a specific inhibitor of IL-27 signaling [79]. An earlier report showed that sIL-27R*α* protects mice from septic peritonitis by neutralizing IL-27, which suppresses the production of reactive oxygen intermediates in endotoxin-stimulated granulocytes and macrophages [80].

Also, the expression levels of gp130 regulate IL-27 signaling. For example, in memory T cells, the downregulation of gp130 expression results in unresponsiveness to IL-27 [81]. The relative levels of STAT1 and STAT3 phosphorylation in response to IL-27 differ in accordance with the cell type and its functional or differentiation status. Indeed, in naïve B-lymphocytes, IL-27 mediates both STAT1 and STAT3 activation, while it triggers moderate STAT1 and low STAT3 activation in memory B cells [82]. Besides STAT1 and STAT3, IL-27 can also signal through STAT5 activation in lung epithelial cells [83]. Finally, the IL-27 receptor is upregulated in intestinal epithelial cells during inflammatory conditions, and IL-27 activates STAT1, STAT3, STAT6, ERK and p38 MAPKs, and Akt, resulting in enhanced cell proliferation [84].

## 3. Role of IL-27 in the Immune Response and Immune-Mediated Disorders

IL-27 is produced during the innate phase of the immune response and regulates the quality and size of the adaptive immune response. Here, we will briefly summarize the role of IL-27 in modulating the immune response, which has been thoroughly addressed elsewhere [47, 85].

### 3.1. Immune-Enhancing Activities of IL-27

Early reports showed that IL-27 is produced by APCs at early stages during antigen-mediated activation and promotes clonal expansion of naïve CD4^+^ T cells. Also, IL-27 mediated Th1 polarization and IFN-*γ* production in naïve CD4^+^ T cells, acting in cooperation with IL-12 [52, 86]. IL-27 induced T-bet and IL-12R*β*2 expression in CD4^+^ T cells, at early phases of Th1 cell polarization, before the action of IL-12 [87]. IL-27 increased mouse CTL generation and proliferation, thus supporting CTL-mediated antitumor effects [34]. Indeed, it upregulated IL-12R*β*2, granzyme B, and perforin expression in anti-CD3-stimulated murine CD8^+^ T cells. In addition, it cooperated with IL-12 in the stimulation of CD8^+^ T cell proliferation and IFN-*γ* production. These effects of IL-27 on naïve CD8^+^ T cells were in part T-bet-dependent [88]. IL-27R is essential for IFN-*γ* production by CD8^+^ T cells during infection in mice [89]. Similarly, IL-27 supported Eomesodermin (Eomes), IFN-*γ*, and granzyme B expression and proliferation in human, CD3-activated CD8^+^ naïve T cells, and increased CTL activity [90]. IL-27 also promotes IL-10 production by mouse CD8^+^ T cells [91], and IL-10 is required for the development of CD8^+^ memory T cells, which may also acquire stemness-like features [92]. In the primary response to a respiratory virus, CD8^+^ T cells secrete IL-10, which is required for the survival of the infected host. However, CD8^+^ T cells fail to release IL-10 during a recall response due to downregulation of gp130 expression, which causes unresponsiveness to IL-27 [81].

### 3.2. Immune-Regulatory and Anti-Inflammatory Properties

IL-27 has anti-inflammatory and immune-regulatory functions and inhibits Th2, innate lymphoid cell-2 (ILC2), and Th17 responses, thus limiting some immune-mediated diseases. In this respect, a first report showed that IL-27 inhibited the expression of GATA-3, which is the Th2-specific transcription factor that impairs Th1 development by downregulation of STAT4, in murine CD4^+^ T cells [86]. Further studies showed that IL-27 downregulated type-2 responses and inhibited CD4^+^ T cell Th2 cytokine production, independently from Th1-related cytokines [93]. In addition, experimental allergic asthma was exacerbated and production of Th2 cytokines enhanced in the lung in WSX1-deficient mice, relative to WT [94]. IL-27 inhibits not only Th2 cell development but also the production of Th2 cytokines in already polarized Th2 cells by altering the GATA-3/T-bet balance. Intranasal treatment with IL-27 inhibited airway inflammation in an asthma model [95]. In spite of an existing body of literature supporting a protective role of IL-27 against Th2 and asthma, a recent report suggested divergent effects in human asthma. Indeed, bronchoalveolar lavage showed increased IL-27 levels in asthmatic patients. In addition, coexpression of IL-27 with CCL26 mRNA, which is prototypical of a Th2/IL-13 signature, was associated with severe asthma [96].

Several studies indicate that IL-27 has anti-inflammatory properties, which limit the severity of autoimmune disorders and regulate immunopathology during infections. These properties are due to the suppression of Th17 cells, the induction of type-1 regulatory (Tr1) T cells, and the upregulation of PD-L1 [reviewed in [97, 98]]. For example, during chronic infection with* Toxoplasma gondii*, WSX1-deficient mice developed exacerbated neuroinflammation that was related to a high IL-17 response. Treatment of naïve T cells with IL-27 inhibited the Th17 cell differentiation induced by IL-6 and TGF-*β* [99]. The suppressive effect of IL-27 on IL-17 production requires STAT1 signaling, which inhibits the expression of the Th17-specific transcription factor ROR*γ*T and may therefore inhibit Th17-mediated DTH responses and EAE [100].

IL-27 stimulates CD4^+^ T cells to express the immune-regulatory cytokine IL-10 [101–103]. Indeed, IL-27 supports the development of immune-regulatory Tr1 CD4^+^ T cells that do not express the FoxP3 transcription factor and regulate T cell function through IL-10 production [104]. DC exposed to classical Treg cells induced the differentiation of Tr1 cells through the IL-27 and TGF-*β* combined action [101]. In mice, IL-27 mediates Tr1 cell expansion and differentiation through the concerted action of the transcription factor c-Maf, IL-21, and the costimulatory receptor ICOS [105].

IL-27 inhibits the production of IL-2 by CD4^+^ Th cells through the induction of SOCS3, thus limiting T cell responses [106, 107]. A recent report indicated that a subset of regulatory CD4^+^ T cells produce IL-27 in response to malaria antigen recognition during infection. In turn, IL-27 inhibits IL-2 production and T cell clonal expansion, thus limiting parasite clearance by the immune system [69].

An additional immune-regulatory mechanism triggered by IL-27 is related to PD-L1 expression. Exposure to IL-27 during the priming of naïve T cells mediates their conversion into regulatory-type T cells, which express PD-L1, through a STAT1-dependent mechanism. In vivo transfer of these cells inhibited Th17 cell development and reduced the severity of autoimmune encephalomyelitis [108]. Also, IL-27 induced PD-L1 expression in human monocyte-derived DC [109] or in mouse liver plasmacytoid DC [110], which then display reduced antigen presentation capabilities. Moreover IL-27-treated plasmacytoid DC increased the percentage of FoxP3^+^ Treg cells in MLR. In mouse DCs, IL-27 also induced CD39 ecto-ATPase expression, which inhibited the generation of effector T cells and the development of EAE [111].

Finally, IL-27 also induces the expression of the immune suppressive enzyme IDO in human adherent monocytes isolated from peripheral blood in vitro [42] and in human neonatal macrophages, which display regulatory functions on T cell proliferation [112].

## 4. Functional Overlaps between IFNs and IL-27

IL-27 and IFNs share the common usage of the STAT1 signaling pathway, which may explain functional overlaps between the two cytokines. Indeed, it was early recognized that IL-27, similar to IFN-*γ*, induced T-bet and IL-12R*β*2 expression in CD4^+^ T cells through signaling via WSX1 and STAT1 and initiated Th1 responses [77, 86, 87, 113]. These effects required WSX1 and STAT1 but were independent of IFN-*γ* production. Moreover, IL-27 mediates T-bet expression and IgG2a class-switch in mouse B cells activated with anti-CD4 and LPS. These effects also occurred in IFN-*γ*-deficient but not in STAT1-deficient B cells [114].

Very recent data indicate that both IL-27 and type-I or type-II IFNs antagonized the functions of human and mouse ILC2 in a STAT1- and ISGF3-dependent fashion [115, 116]. These cytokines inhibited ILC2 cytokine secretion, cell proliferation, and survival, resulting in reduced Th2 lung immunopathology.

IL-27 has been reported to induce the expression of MHC class-I molecules, a typical effect of IFNs, in different cell types including mouse naïve CD4^+^ T cells [113], neuroblastoma cells [34], human monocytic cell lines [117], endothelial cells [118], hepatocytes, hepatoma cell lines [119], and hepatic stellate cells [120]. Moreover, IL-27 upregulated MHC class-II molecules during mouse monocytic DC cell differentiation [121] and in human endothelial cells [118].

Besides the common effects on lymphoid cell functions and MHC molecule upregulation, IL-27 displays IFN-like antiviral properties. Indeed, IL-27 is a potent inhibitor of HIV-1 replication in PBMCs, CD4^+^ T cells, and macrophages [122]. A further study showed that IL-27 activates several IFN-inducible genes such as myxovirus protein, oligoadenylate synthetase, RNA-dependent kinase, and apolipoprotein B mRNA-editing enzyme-catalytic polypeptide-like (APOBEC)-3G, in human monocyte-derived macrophages [123]. These effects were independent of IFNs. The IFN-inducible transcription factor BST-2 was required for APOBEC-3 gene activation by IL-27, in a similar fashion as IFN-*γ* [124].

An analysis of IL-27-induced gene expression in human macrophages showed a broad overlap of antiviral gene induction, similar to that of IFN-*α* [125]. IL-27 inhibited hepatitis C virus (HCV) replication in an HCV-permissive cell line, through the activation of IFN-inducible antiviral genes, in an IFN-independent manner [126].

We previously showed that IL-27, similar to IFN-*γ*, upregulated the expression of IL-18BP, a natural inhibitor of IL-18 activity, in ovarian cancer cells, and that IL-18BP protein accumulates in the ascites of patients [43]. A similar IL-18BP-induction by IL-27 was also recorded in human keratinocytes [127], and in both reports this effect was STAT1-mediated. Also, IL-27 mediated IDO and PD-L1 expression in ovarian cancer cells, suggesting that the effect of IL-27 and IFN-*γ* may show a broader overlap [42]. This hypothesis was recently confirmed by a proteomic analysis of IFN-*γ* or IL-27-treated ovarian cancer cells, which showed that 82.2% of modulated proteins were concordantly regulated by the two cytokines. Indeed, bioinformatics analyses of IL-27-regulated pathways revealed induction of proteins involved in interferon signaling and regulation, HLA class-I antigen presentation, protection from natural killer cell-mediated cytotoxicity, antiviral activity, regulation of proteasome function, and amino acid catabolism [25]. We found that IL-27 enhanced HLA class-I molecule levels in different types of human cancer cells, including neuroblastoma tumor cells, which showed very low constitutive expression [128]. Altogether, these data indicate that IL-27 and IFN-*γ* display a broad overlap of functions in human cancer cells, which are related to STAT1 pathway activation by both cytokines. Therefore, the effects of STAT1 seem to prevail over those of STAT3 signaling, which is also activated by IL-27 and may be involved in the regulation of a smaller set of proteins. Consistent with these data, a very recent gene expression study reported that IL-27 responses were most similar to STAT1-dominated, IFN-*γ*-mediated responses, while they differed from STAT3-dominated, IL-6-type cytokine-mediated ones, in hepatocellular carcinoma cells [129]. However, in STAT1 gene-silenced hepatocellular carcinoma cells, IL-27 mediated expression of *γ*-fibrinogen, which is typically induced by IL-6. Moreover, IL-6 prestimulation inhibited the cellular responsiveness to IL-27 but not to IFN-*γ*, possibly through a STAT3/SOCS3-mediated mechanism. The authors conclude that IL-27 shows transcriptional effects broadly overlapping with those of IFN-*γ* in hepatic cells, although only the IL-27 response is sensitive to SOCS3 inhibition [129].

## 5. Role of IL-27 in Cancer

**Figure 2:**
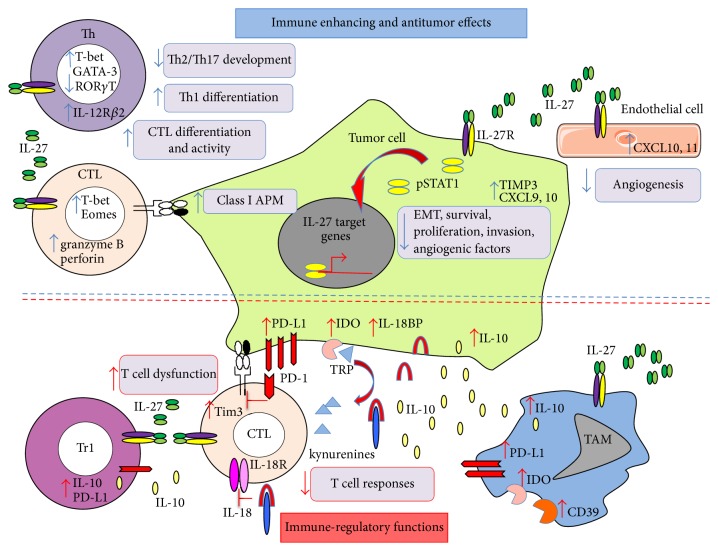
Summary of immune-enhancing and antitumor versus immune-regulatory and potentially protumor effects of IL-27. Blue arrows (upper half of figure) indicate immune-enhancing and antitumor effects, whereas red arrows (lower half) represent immune-regulatory functions.

Besides its effects on HLA class-I induction in human cancer cells, IL-27 has shown antitumor activity in several tumor models in vitro and in vivo. IL-27 can act through multiple mechanisms such as activation of antitumor immune responses and direct inhibition of tumor cell proliferation, survival, and angiogenic and invasive properties [29, 49]. Nonetheless, the effects of IL-27 on the immune response may be dual, resulting in tumor promoting effects in vivo, as suggested by increased IL-27 expression in some human cancers. In addition, IL-27 may drive the expression of different immune-regulatory molecules in human cancer cells, which may support local derangement of the immune response in vivo, as summarized in Figure 2.

### 5.1. Antitumor Activities of IL-27

Several reports indicate that IL-27 may exert direct inhibitory effects on tumor cells expressing the WSX1/gp130 receptor. For example, IL-27 treatment decreased proliferation and expression of angiogenesis and invasion-related genes in human pediatric acute myeloid leukemia (AML) cells in vitro. Moreover, in NOD/SCID/Il2r*γ* −/− mice, it suppressed AML cell expansion, survival, and invasive properties [21]. Similarly, IL-27 inhibited proliferation and increased apoptosis of human prostate cancer cells [23], multiple myeloma (MM) primary cells and cell lines [24], non-small cell lung (NSCLC) cancer cells [22], and ovarian cancer cell lines [25, 26]. In NSCLC, IL-27 downregulated stemness- and EMT-related genes but also pushed intratumor myeloid cells to exert antitumor effects in xenotransplant models [22]. IL-27 treatment suppressed angiogenesis, osteoclast differentiation, and bone erosive activity, while it supported osteoblast proliferation in human MM cells xenotransplant models [24]. The combined use of the COX-2 inhibitor Apricoxib and IL-27 cooperatively inhibited EMT transition of NSCLC cells in a STAT1-dependent manner [27].

The direct antitumor effects require the expression of WSX1 and STAT1 signaling. Indeed, IL-27 suppressed epithelial-mesenchymal transition and expression of the proangiogenic factors VEGF, CXCL8, and CXCL5 in human NSCLC cells. STAT1 silencing by siRNA reversed the effects of IL-27, whereas a STAT3 inhibitor had no effect [28]. Also, WSX1-negative B16F10 mouse melanoma cells became sensitive to IL-27 antiproliferative activity upon WSX1 transfection, whereas transfection of a mutant WSX1 unable to recruit STAT1 was ineffective [29]. Moreover, human melanoma cells, which constitutively express WSX1, showed growth inhibition by IL-27. Another study reported that inhibition of human melanoma cells by IL-27 involved induction of TRAIL expression. In addition, a combination of IL-27 with the TLR3 ligand poly (I:C) cooperatively suppressed melanoma growth in xenograft models [30].

Besides its direct inhibitory activity on tumor cells, IL-27 may also exert indirect antitumor effects by targeting the tumor microenvironment. IL-27 expressed by gene transfer in B16F10 melanoma may exert antiangiogenic functions on subcutaneous tumors and lung metastases. These effects did not require IFN-*γ* and a functional immune system, as they were also evident in IFN-*γ*-deficient mice and NOD/SCID mice. IL-27 may directly suppress angiogenesis in different experimental models and induce production of antiangiogenic chemokines CXCL9 and CXCL10 by endothelial cells in vitro. Also, the antitumor activity of IL-27 was partially inhibited by an anti-CXCL10 antibody in the B16F10 melanoma model [31]. Similarly, IL-27 induced CXCL9 and CXCL10 gene expression in primary human umbilical vein endothelial cells through a STAT1 involving mechanism [32].

Several reports indicated a significant role of the immune-enhancing activities in the IL-27-mediated antitumor effect through the upregulation of Th1 and CTL responses. For example, mouse TBJ neuroblastoma [34] or C26 colon carcinoma cells [33], genetically engineered to express a single-chain IL-27 molecule, lost their tumorigenic potential when implanted in syngeneic mice. Mice that eventually rejected IL-27-expressing cells developed immunity to tumor antigens as demonstrated by rechallenge experiments with wild-type tumor cells. These effects were strictly dependent on CD8^+^ CTLs and production of IFN-*γ* in both models. In the C26 model, the Th1-related transcription factor T-bet, but not STAT4, was strictly required for IL-27 antitumor activity, strongly supporting a role of a Th1 response in the antitumor effect [33]. In the TBJ neuroblastoma model, the effect of transduced IL-27 could be further enhanced by the administration of IL-2, which led to the complete regression of neuroblastoma metastases [35].

Other reports indicated that IL-27 mediated the activation of NK cell responses, which may result in antitumor effect in vivo. Indeed, IL-27 induced T-bet and perforin and increased the cytolytic activity of murine DX5^+^ NK cells in vitro. IL-27 therapy inhibited the growth of NK-resistant head and neck squamous carcinoma cells in syngeneic mice through the induction of an anti-tumor IgG antibody response and NK-mediated antibody-dependent cellular cytotoxicity [36]. In addition, human oesophageal carcinoma cells expressing IL-27 showed decreased growth when implanted in nude mice, possibly through enhanced NK cell activity and IFN-*γ* production [37].

Recent data suggest that IL-27 may inhibit the M2 macrophage polarization of the human promonocytic cell line U937 and the proliferation and migration of cocultured pancreatic tumor cells. Therefore, the authors proposed that IL-27 could be tested to revert M2 polarization of pancreatic tumor-associated macrophages [38]. In NSCLC patients, serum IL-27 levels are reduced and negatively correlated with the Th17 cell counts in the peripheral blood and ROR*γ*T mRNA expression. These data suggest that IL-27 might inhibit the development of Th17 cells, which may be regarded as a potential target in NSCLC [130].

Reports in KO models suggest a role for endogenous IL-27 in the control of tumor growth. A study assessed spontaneous tumorigenesis in WSX1 deficient mice bred with inactive-mutant p53 mice, which mimic the Li-Fraumeni syndrome. WSX1(−/−)mice, homozygous or heterozygous for mutant p53, showed more rapid tumor development and shorter survival than their WSX1(+/+) counterparts. Also, in mutant p53 heterozygous mice, the absence of WSX1 enhanced the incidence of osteosarcomas. Therefore, the lack of IL-27 signaling modulates the effects of p53 mutations in vivo and enhances spontaneous carcinogenesis [39]. Similarly, EBI3-deficient BALB/c or C57BL/6 mice showed reduced antitumor immune responses and enhanced tumor growth when challenged with J558 plasmacytoma or B16 melanoma cells, respectively [40]. The presence of functional Treg cells, expressing high levels of IL-10, suggests that the lack of EBI3 results in a dominant IL-27-deficient phenotype rather than an IL-35 deficient one, which would result in enhanced antitumor immunity. A recent study showed that IL-35, produced by intratumor Treg cells, limits antitumor T cell immunity. Indeed IL-35 induces the expression of inhibitory receptors such as PD1, TIM3, and LAG3, which mediate T cell dysfunction in the tumor environment [131].

Nonetheless, the interpretation of data obtained in KO mouse models of IL-27 or IL-27R chains may be complicated due to the two-chain structure and promiscuous usage of chains among the different members of the IL-27 and IL-6 families of cytokines and their receptors. For example, the results of studies obtained in EBI3-defective mouse strains may not be unambiguously assigned to IL-27-deficiency, as the EBI3 chain may be secreted as a monomer or can form dimers with IL-12p35 to form IL-35 or with IL-23p19 to form IL-39.

### 5.2. Tumor-Promoting Activities of IL-27

Although IL-27 has shown well-documented antitumor activity in several experimental models, a few reports suggest that it may also have potential tumor-promoting effects. For example, IL-27 serum levels are elevated in gastroesophageal cancer, in a relationship with the lymph node involvement [132], and in breast cancer patients in correlation with VEGF and clinical stage [133]. In addition, different from the IL-27 heterodimer, which inhibits prostate cancer cell proliferation and survival [23], IL-27p28 (IL-30) has shown a protumorigenic role in both human prostate and breast cancer. Indeed, high IL-30 expression has been reported in human prostatic cancer cells, macrophages, and other myeloid cells in the prostatic tumor microenvironment. Importantly, IL-30 expression correlated with tumor stage and grade. IL-30 also stimulated the proliferation of PC3 prostate cancer cells which coexpress gp130 and IL-6R*α*, in vitro, supporting a potential protumor role of IL-27p28 in prostate tumors [134]. In breast cancer, high IL-30 expression levels by tumor and draining lymph node infiltrating monocytes, macrophages, and CD33^+^/CD11b^+^ myeloid cells correlated with triple-negative and HER2^+^ molecular types, advanced stage, recurrence, and reduced overall survival. Moreover, IL-30 upregulated IL-6 expression, proliferation, and migration of breast cancer cells through STAT1/STAT3 signaling and supported the growth of triple-negative breast cancer xenografts [135].

Opposite to the findings in pediatric AML [21], IL-27 promoted the proliferation and survival of adult AML cell lines coexpressing WSX1 and gp130. Consistently, IL-27 decreased TNF-*α*-induced apoptosis and the responsiveness to cytarabine and daunorubicin. IL-27 activated the STAT1/3 and ERK1/2 pathways in the leukemic cells, and IL-27 proliferative effects were blocked by the MEK inhibitor U0126 [41].

Moreover, IL-27 is highly expressed in invasive cutaneous melanoma, particularly at advanced stages of progression, whereas no expression was found in benign nevi and in situ melanomas. IL-27 expression correlated with that of PD-L1 and IL-10 in melanoma samples, and IL-27 induced IL-10 and PD-L1 expression in melanoma cells in vitro [136]. Similarly, IL-27 was found to induce the expression of immune-regulatory molecules such as IL-18BP, the natural inhibitor of IL-18, and PD-L1 and IDO in human ovarian cancer cells [42, 43] and also in other cancer cells in vitro. Induction of surface PD-L1 expression was predominantly STAT3-dependent, whereas STAT1 was required for IDO mRNA and protein induction in ovarian cancer cells. Expression of IL-27p28 and EBI3 was also detected in tumor- or ascites-associated leukocytes with myeloid features. In addition, cells isolated from ascites showed constitutive STAT1 and STAT3 tyrosine phosphorylation and IDO expression. Altogether, these data suggested a possible immune-regulatory role of endogenous IL-27 in ovarian cancer [42].

Besides the activity on tumor cells, IL-27 may also induce an immune-regulatory phenotype in tumor-associated macrophages through upregulation of CD39 or PD-L1. Indeed, CD14^+^CD163^+^ tumor-associated macrophages from ovarian cancer patients or macrophages generated by M-CSF stimulation in vitro display high surface expression of the ectonucleotidase CD39. A CD39 inhibitor reduced the immune-regulatory activity of these macrophages and their ability to release IL-10. IL-27, produced by tumor-associated neutrophils, may induce CD39 expression and immune-regulatory activity in macrophages [44]. Moreover, macrophages infiltrating human lymphoma tissues such as adult T cell lymphoma/leukemia, follicular lymphoma, and diffuse large B cell lymphoma express PD-L1 [45]. Lymphoma cell line supernatant or IL-27 induced PD-L1/2 expression in human macrophages through a STAT3-dependent mechanism. However, human lymphoma cells express high EBI3 but not IL-27p28 [45, 137]. It has been proposed that EBI3 produced by lymphoma cells may pair with macrophage-released IL-27p28 to form the IL-27 heterodimer, which induces PD-L1/2 expression [45]. IL-27 also induces the expression of the inhibitory receptor T cell immunoglobulin and mucin domain-3 (Tim-3), a potent inducer of T cell dysfunction. This effect requires the IL-27-mediated induction of the nuclear factor, interleukin 3 regulated (NFIL3), which mediates Tim-3 expression in vivo. B16F10 melanoma or Lewis lung carcinoma implanted into WSX1−/− mice exhibited slower tumor growth than WT littermates. Moreover, TILs from WSX1−/− mice displayed reduced expression of NFIL3, Tim-3, and PD-1, which are markers of exhausted T cells. CD8^+^ T cells from WSX1−/− mice produced more IL-2, IFN-*γ*, and TNF than CD8^+^ T cells from control mice. These data support a role for endogenous IL-27 as an inducer of T cell dysfunction through the induction of Tim-3 and IL-10 [46].

## 6. Concluding Remarks and Perspectives for Immunotherapy

IL-27 may have a therapeutic potential in human cancer therapy as suggested by a significant number of mouse preclinical tumor models, which showed a potent antitumor activity (Table 1). In most instances, this activity relates to the activation of Th1 and CTL antitumor responses, which lead to tumor regression. In human tumor cells, IL-27's biological activities show a broad overlap with those of IFN-*γ*, including the upregulation of the HLA class-I antigen presentation machinery, which may favor CTL-mediated tumor cell recognition. This property may be particularly relevant for immunotherapy of tumors with downregulated MHC class-I molecule expression such as neuroblastoma [25]. Also, IL-27 may exert direct inhibitory effects on human tumor cells through the inhibition of their proliferation, survival, invasiveness, and proangiogenic properties. IL-27-sensitive tumors include solid cancers (e.g., NSCLC, prostate cancer, ovarian cancer, and melanoma) [22, 23, 25–27, 29, 30] and hematologic malignancies (e.g., pediatric AML and myeloma) [21, 24]. These tumors may represent optimal targets for exploiting both the direct antitumor and immune-enhancing activities of IL-27 in future clinical studies.

However, IL-27 has also well-known immune-regulatory functions, such as induction of immune-regulatory Tr1 cells and upregulation of immune suppressive molecules, including IL-10, TIM-3, IDO, CD39, and PD-L1. Therefore, IL-27 may have a similar role as IFN-*γ* in the induction of immune resistance of tumors, as suggested by the expression of IL-27 in the microenvironment of certain tumors. Also, IL-27 levels have been found to be elevated in some cancer patients [132, 133], thus indicating potential protumor effects of endogenous IL-27 in cancer progression (Table 1). Altogether, these findings suggest a dual role of IL-27 in tumor immunology, which may limit its applications in cancer immunotherapy. Nonetheless, one may speculate that the immune-enhancing and direct antitumor effects of IL-27 may be best exploited in combination with agents that limit IL-27-induced immune-regulatory mechanisms, for example, IDO inhibitors or anti-PD-L1/PD-1 antibodies.

On the other hand, IL-27 may find applications related to its immune-regulatory and anti-inflammatory properties. In particular, its ability to enhance IL-10 and PD-L1 expression and inhibit ROR*γ*T-dependent Th17 cell development [100] suggested that IL-27 may be considered as a potential therapeutic agent for some autoimmune or inflammatory diseases. This hypothesis has been supported by the study of animal models, for example, colitis [138], multiple sclerosis [100, 108, 111], or rheumatoid arthritis [139]. Nonetheless, IL-27 may display pro- or anti-inflammatory activity in relationship with the different tissues involved, the type and phase of autoimmune disease, or the underlying effector mechanism (discussed in [98, 140]). The ability of IL-27 to inhibit GATA-3 expression and Th2 and ILC2 development [86, 93, 94, 115, 116] suggested a potential usage of IL-27 in severe asthma, based on preclinical models [95]. Conversely, IL-27 may have proinflammatory and pathogenic role in other conditions, where the usage of IL-27 blocking agents, such as monoclonal antibodies or a soluble WSX1 [79], may be useful. This might be the case of Crohn's disease, where Th1 responses have a pathogenic role and high expression of IL-27 has been reported [141].

In conclusion, IL-27 may be regarded as a new tool or target for manipulating the immune system in different immune-mediated diseases and in cancer. However, the pleiotropic functions of this cytokine and its dual role in immune regulation should be considered in the design of clinical trials, which have been planned but not yet initiated.

## Figures and Tables

**Table 1a:** (a) Antitumor effects

Target cells or models	Major effects	Ref.^a^
*Direct antitumor effects*		
AML cells implanted in NOD/SCID/Il-2r*γ*−/− mice	IL-27 suppressed human pediatric AML cell expansion, survival, and invasive properties	[21]
NSCLC cells in xenotransplant models	IL-27 downregulated stemness and EMT genes in human NSCLC cells and activates intratumor myeloid cells to exert antitumor effects	[22]
PC3 or DU145 human prostate cancer cell injection in athymic nude mice	IL-27 treatment reduced proliferation and vascularization in association with ischemic necrosis of tumors	[23]
Human multiple myeloma xenotransplant models	IL-27 treatment suppressed angiogenesis, osteoclast differentiation, and bone erosive activity, while it supported osteoblast proliferation	[24]
Human ovarian cancer, neuroblastoma	IL-27 induced HLA class-I antigen presentation machinery component expression and surface HLA class-I molecules and inhibited survival and migration of ovarian cancer cells	[25]
SKOV3 human ovarian cancer cell line	The overexpression of IL-27 enhanced cell death and inhibited the proliferation of SKOV3 cells	[26]
Human NSCLC cells	The combined use of the COX-2 inhibitor Apricoxib and IL-27 cooperatively inhibited EMT transition	[27]
Human NSCLC cells	IL-27 suppressed epithelial-mesenchymal transition and expression of proangiogenic factors	[28]
Mouse melanoma B16F10 cell transfectants expressing wild-type WSX1	IL-27 showed antiproliferative activity on melanomas through WSX1/STAT1 signaling	[29]
Human melanoma in immunodeficient mice	Combination of IL-27 with the TLR3 ligand poly (I:C) cooperatively suppressed melanoma growth	[30]

*Indirect antitumor effects by targeting the tumor microenvironment*		
B16F10 mouse melanoma model	IL-27 induced production of antiangiogenic chemokines CXCL9 and CXCL10 by endothelial cells	[31]
Primary endothelial cells	IL-27 induced CXCL9 and CXCL10 gene expression	[32]
C26 colon carcinoma cells transduced with the single-chain IL-27 cDNA	IL-27-dependent tumor-specific activity and protective immunity are mediated mainly through CD8^+^ CTLs and production of IFN-*γ*	[33]
Mouse TBJ neuroblastoma cells engineered to overexpress IL-27	TBJ-IL-27 tumors showed enhanced IFN-*γ* and MHC class-I expression in conjunction with tumor-specific CD8^+^ CTL reactivity. IL-27 and IL-2 cooperated in inducing regression of metastases	[34, 35]
Head and neck squamous cell carcinoma and IL-27 gene transfer in syngeneic mice	IL-27 induced T-bet and perforin in NK cells. It inhibited the growth of NK-resistant tumors through induction of NK-mediated ADCC	[36]
Eca109 human oesophageal carcinoma cells expressing IL-27 in nude mice	Tumor growth was retarded in vivo, possibly through enhanced NK cell activity and IFN-*γ* production	[37]
Human promonocytic cell line U937	IL-27 inhibited the M2 macrophage polarization	[38]
IL27RA(−/−) mice bred with mutant p53 heterozygous (p53(R172H/+)) mice	More rapid spontaneous tumor development and reduced survival of IL27RA(−/−)p53(H/+ or H/H) mice relative to their WSX1(+/+) counterparts	[39]
J558 plasmacytoma or B16 melanoma injected in EBI3-deficient BALB/c or C57BL/6 mice, respectively	Reduced antitumor immune responses and enhanced tumor growth relative to wild-type control mice. Tumors from EBI3-deficient mice contained significantly decreased proportions of CD8^+^ T cells and increased proportions of CD4^+^FoxP3^+^ Treg cells	[40]

^a^Only selected references are reported.

**Table 1b:** (b) Protumor effects

Target cells or models	Major findings	Ref.
OCI-AML5 acute myeloid leukemia and TF-1, UT-7, and UT-7/EPO erythroleukemic cell lines	IL-27 promoted survival, reduced TNF-*α*-induced apoptosis, and decreased the responsiveness of adult AML cells to cytarabine and daunorubicin	[41]
Human ovarian cancer cells	IL-27 induced the expression of immune-regulatory molecules such as IL-18BP, PD-L1, and IDO in human ovarian cancer cells	[42, 43]
Human ovarian cancer-associated macrophages	IL-27 induced CD39 expression and acquisition of immune-regulatory activity	[44]
Human lymphoma macrophages	IL-27 induced PD-L1/2 expression through a STAT3-dependent mechanism	[45]
Mouse tumor-associated T lymphocytes	IL-27 induced the expression of Tim-3, a potent inducer of the T cell dysfunction, and IL-10	[46]
